# Prevalence of corneal astigmatism in patients undergoing cataract surgery in India

**DOI:** 10.6026/973206300214516

**Published:** 2025-12-15

**Authors:** Mousmi Anand, Sunil Kumar, Deepankar Deepankar, Neda Rahman, Rohit Kumar Mahato

**Affiliations:** 1 Regional Institute of Ophthalmology, Rajendra Institute of Medical Sciences, Ranchi, Jharkhand, India

**Keywords:** Corneal astigmatism, cataract surgery, keratometry, corneal topography, refractive error, visual outcomes

## Abstract

Corneal astigmatism is common among patients presenting for cataract surgery. Hence, understanding its pattern is essential for optimal surgical planning and visual outcomes. This study investigated the prevalence, type, and severity of corneal astigmatism in 320 cataract surgery candidates aged 40 years and above at RIO, RIMS, Ranchi, India. Automated keratometry and corneal topography were used to classify astigmatism by orientation and magnitude. Corneal astigmatism was present in 89.4% of patients, with 68.1% showing clinically significant levels (≥1.0 D), most commonly with-the-rule astigmatism, followed by against-the-rule and oblique types. These findings highlight the importance of thorough preoperative astigmatism assessment and appropriate corrective planning to ensure optimal postoperative visual outcomes.

## Background:

Cataract surgery has evolved from a vision-restoring procedure to a refractive procedure aimed at achieving excellent uncorrected visual acuity and reducing dependence on spectacles [1]. With advancing surgical techniques and premium intraocular lens (IOL) options, patient expectations for optimal post-operative vision have increased significantly. However, pre-existing corneal astigmatism remains one of the major barriers to achieving these goals, as uncorrected astigmatism can significantly compromise visual quality even after successful cataract extraction [2]. This condition affects approximately 95% of the general population to some degree, with clinically significant astigmatism (≥1.0 diopters) present in 20-40% of individuals depending on the population studied [3, 4]. The prevalence and characteristics of corneal astigmatism vary significantly across different geographical regions, ethnicities, and age groups, making population-specific studies essential for surgical planning. The classification of corneal astigmatism is based on the orientation of the steepest meridian. With-the-rule (WTR) astigmatism occurs when the vertical meridian is steeper than the horizontal meridian (steep axis between 60° to 120°), against-the-rule (ATR) astigmatism when the horizontal meridian is steeper (steep axis between 0° to 30° or 150° to 180°), and oblique astigmatism when the steep axis lies between 30° to 60° or 120° to 150° [5]. Age-related changes in corneal curvature typically show a shift from WTR astigmatism in younger individuals to ATR astigmatism in older adults, which is particularly relevant in cataract surgery patients [6]. The magnitude of corneal astigmatism is equally important in surgical planning. Low astigmatism (0.5-1.5 D) may be managed conservatively or with minor surgical modifications, moderate astigmatism (1.5-3.0 D) often requires specific interventions such as toric IOLs or limbal relaxing incisions, while high astigmatism (>3.0 D) may necessitate more complex surgical approaches including corneal refractive procedures [7, 8]. Accurate pre-operative assessment of corneal astigmatism has become increasingly sophisticated with the advent of advanced diagnostic tools. Manual keratometry, automated keratometry, and corneal topography each provide valuable information about corneal curvature, with topography offering the most comprehensive analysis of corneal surface irregularities [9]. These measurements are crucial for IOL power calculations, toric IOL selection, and planning of astigmatism correction procedures. However, toric IOLs have emerged as the most predictable method for correcting moderate to high astigmatism, providing excellent visual outcomes when properly selected and positioned [10, 11]. The success of these interventions depends heavily on accurate pre-operative assessment and understanding of the astigmatism characteristics in the specific population. Even though corneal astigmatism plays a big role in how well cataract surgery turns out, we don't have much data on how common it is or what it looks like in Eastern India, especially in Jharkhand. Knowing the specific patterns of astigmatism in our patients is key to planning the right surgical approach, choosing the best intraocular lens (IOL), and boosting overall results.

## Study design:

We carried out this year-long study at the Regional Institute of Ophthalmology (RIO), part of the Rajendra Institute of Medical Sciences (RIMS) in Ranchi, from January 2023 to December 2023. We designed it as a prospective observational study to gather detailed information on how common corneal astigmatism is and what its characteristics are among local patients preparing for cataract surgery.

## Study population:

We included patients 40 years and older who were lined up for cataract surgery at the Regional Institute of Ophthalmology (RIO), part of the Rajendra Institute of Medical Sciences (RIMS) in Ranchi. After applying our inclusion and exclusion criteria, we enrolled a total of 320 patients. To figure out the sample size, we used a formula for prevalence studies, estimating that about 30% of patients would have corneal astigmatism, with a 5% margin of error and a 95% confidence level, which gave us a minimum needed sample size of 323 patients.

## Inclusion criteria:

We included patients who were 40 or older and scheduled for cataract surgery, had visually significant cataracts (with vision of 6/60 or worse or noticeable visual symptoms), were willing to join the study and give informed consent, and had clear corneas that allowed for accurate keratometric measurements.

## Exclusion criteria:

We excluded patients who had active eye infections or inflammation, had undergone previous eye surgeries (including refractive surgery), or had corneal issues like scars or dystrophies that could mess with keratometric measurements. We also left out those with irregular astigmatism or keratoconus, anyone who couldn't cooperate during exams, and those with incomplete data or who didn't follow up.

## Methodology:

Every patient received a thorough eye check-up, which included a detailed medical history, vision testing with a Snellen chart, slit-lamp biomicroscopy, and a dilated fundus exam. To assess their corneal astigmatism, we used both automated keratometry (with the IOL Master 500 from Carl Zeiss Meditec) and corneal topography (using the Pentacam HR from Oculus) to ensure our measurements were accurate and reliable. Trained technicians took at least three readings per eye under consistent conditions, and we used the average values for our analysis.

## We grouped corneal astigmatism by severity:

Low (0.5-1.5 diopters), moderate (1.5-3.0 diopters), and high (over 3.0 diopters). Classification of the astigmatism relied on the axis orientation: it was deemed with-the-rule (WTR) for a steep axis ranging from 60° to 120°, against-the-rule (ATR) for ranges of 0°-30° or 150°-180°, and oblique for ranges of 30°-60° or 120°-150°. We considered astigmatism clinically significant if it was 1.0 diopters or more, as this level can noticeably affect vision and may need correction during cataract surgery. We also collected other details like the patients' age, gender, occupation, which eye was affected, the type and severity of their cataracts (using the Lens Opacities Classification System III, or LOCS III), and any other optical conditions they had. To keep our data reliable, we regularly calibrated our equipment, followed standardized exam protocols, and double-checked all recorded information.

## Statistical analysis:

We collected the data and analysed it using Python. We used statistics to describe the patients and their astigmatism. For continuous variables, such as age, we calculated means and standard deviations, while for categorical variables, like astigmatism types, we reported counts and percentages. To examine associations between categorical variables, we applied the Chi-square test, and for comparing continuous variables across groups, we used the t-test. Additionally, we conducted correlation analyses to investigate relationships between age, gender, and astigmatism parameters. A p-value less than 0.05 were deemed statistically significant.

## Results:

We studied 320 patients scheduled for cataract surgery, analysing just one eye per patient (320 eyes total) to avoid any bias from including both eyes. Below, we break down the demographic details and patterns of corneal astigmatism we found. The average patient age was 64.2 years (SD 8.7), ranging from 42 to 84 years, with 62.5% aged 60-75. There were 182 men (56.9%) and 138 women (43.1%), with a male-to-female ratio of 1.3:1. We examined the right eye in 168 patients (52.5%) and the left eye in 152 (47.5%). Most patients (78.4%) were from rural areas, fitting our tertiary care centre's location. Corneal astigmatism (≥0.25 diopters) was present in 286 patients (89.4%), while 34 (10.6%) had minimal or no astigmatism. Clinically significant astigmatism (≥1.0 diopters) affected 218 patients (68.1%), and 156 (48.8%) had moderate to high astigmatism (≥1.5 diopters) (Table 1 - see PDF). This shows corneal astigmatism is common, with over two-thirds needing correction during cataract surgery. Most patients (51.2%, 164 people) had low astigmatism (0.5-1.5 diopters). Moderate astigmatism (1.5-3.0 diopters) affected 32.8% (105 people), and high astigmatism (over 3.0 diopters) was seen in 16.0% (51 people). The average corneal astigmatism was 1.8 ± 1.2 diopters overall, and 2.0 ± 1.1 diopters for those with measurable astigmatism. The classification of corneal astigmatism based on axis orientation revealed interesting patterns as shown in Table 2 (see PDF). With-the-rule (WTR) astigmatism was the most prevalent type, present in 137 patients (42.8%), followed by against-the-rule (ATR) astigmatism in 101 patients (31.6%), and oblique astigmatism in 82 patients (25.6%). This distribution differs from some Western populations where ATR astigmatism is more common in older adults.

Analysis of corneal astigmatism patterns across different age groups revealed significant age-related changes as demonstrated in Table 3 (see PDF). In younger patients (40-60 years), WTR astigmatism was most common (58.7%). In older patients (>70 years), ATR astigmatism was more frequent (41.8%). This age-related change was statistically significant (p<0.001) and matches known corneal aging trends. Gender-based analysis showed some interesting differences in corneal astigmatism patterns. Males had a slightly higher mean astigmatism magnitude (1.9 ± 1.3 D) compared to females (1.7 ± 1.1 D), although it was not statistically significant (p=0.156). WTR astigmatism was more common in females (46.4%) compared to males (40.1%), while ATR astigmatism showed the opposite trend with higher prevalence in males (34.6%) compared to females (27.5%). The relationship between cataract severity (graded using LOCS III) and corneal astigmatism showed that patients with denser cataracts (grade 3-4) had slightly higher mean astigmatism (2.0 ± 1.4 D) compared to those with less dense cataracts (1.7 ± 1.0 D). However, this correlation was not statistically significant (r=0.124, p=0.089), implying that cataract density does not significantly influence corneal astigmatism measurements in this population. When analysing the clinical significance of the findings, 218 patients (68.1%) had astigmatism ≥1.0 D that would benefit from correction during cataract surgery. Among these, 156 patients (48.8%) had astigmatism ≥1.5 D that would be good candidates for toric IOL implantation or surgical astigmatism correction techniques. The elevated incidence of astigmatism with clinical relevance underscores the critical need for evaluating and strategizing astigmatism correction before surgery in this group of patients.

## Discussion:

This study comprehensively details corneal astigmatism in cataract surgery patients at a major Eastern Indian tertiary care centre. Its findings highlight crucial patterns with significant implications for surgical planning and patient outcomes. The overall prevalence of corneal astigmatism (89.4% with any measurable astigmatism and 68.1% with clinically significant astigmatism) in our study is higher than reported in many Western populations but consistent with some Asian studies [12, 13]. This high prevalence emphasizes the critical importance of routine pre-operative astigmatism assessment in cataract surgery patients in our region. The finding that more than two-thirds of patients have clinically significant astigmatism suggests that astigmatism correction should be considered as a standard part of cataract surgery rather than an optional enhancement. The distribution of astigmatism magnitude shows a predominance of low to moderate astigmatism, with 51.2% having low magnitude (0.5-1.5 D) and 32.8% having moderate magnitude (1.5-3.0 D) astigmatism. This distribution is favourable for surgical correction, as most of these cases can be effectively managed with toric IOLs or surgical techniques like limbal relaxing incisions [14, 15]. The 16% prevalence of high astigmatism (>3.0 D) is notable and may require more complex surgical approaches or staged procedures for optimal correction. The predominance of with-the-rule astigmatism (42.8%) in our study population is somewhat unexpected, as many studies in older adults show a predominance of against-the-rule astigmatism [16, 17]. This finding may reflect ethnic or regional differences in corneal aging patterns, or it could be related to the specific age distribution of our study population. The relatively high prevalence of oblique astigmatism (25.6%) is also noteworthy, as oblique astigmatism is often considered more challenging to correct surgically and may be associated with poorer visual outcomes [18]. The age-related shift from with-the-rule to against-the-rule astigmatism observed in our study is consistent with established patterns of corneal aging. The physiological basis for this shift involves changes in corneal biomechanics, eyelid tension, and extraocular muscle forces with aging [19]. Understanding this pattern is crucial for surgical planning, as against-the-rule astigmatism is generally considered more difficult to correct with certain techniques and may require different surgical approaches. Gender-based differences in our study were relatively minor, with males showing slightly higher mean astigmatism magnitude. Some studies have reported more significant gender differences, possibly related to hormonal influences on corneal structure or occupational factors [20]. The lack of strong gender associations in our study suggests that gender should not be a major factor in astigmatism correction planning, though individual assessment remains essential. The weak correlation between cataract severity and corneal astigmatism in our study is reassuring from a measurement accuracy standpoint. Dense cataracts can sometimes interfere with accurate keratometric measurements, but our findings suggest that reliable corneal curvature assessment is possible in most cataract surgery candidates [21]. This supports the feasibility of routine pre-operative astigmatism assessment even in patients with advanced cataracts. From a clinical management perspective, our findings have several important implications. The high prevalence of clinically significant astigmatism (68.1%) suggests that astigmatism correction should be discussed with most cataract surgery patients. For the 48.8% of patients with moderate to high astigmatism (≥1.5 D), toric IOL implantation represents an excellent option for achieving optimal visual outcomes [22, 23]. The 16% of patients with high astigmatism may benefit from additional procedures such as limbal relaxing incisions or even staged corneal refractive surgery. The economic implications of these findings are also significant. While toric IOLs and astigmatism correction procedures involve additional costs, the high prevalence of clinically significant astigmatism in our population suggests that these interventions could benefit a large number of patients. Cost-effectiveness studies specific to our population would be valuable for healthcare policy decisions. Comparing our findings with international studies shows both consistencies and differences. The overall prevalence of astigmatism in our study is similar to what's reported in other Asian populations, though it's higher than in many European studies [24, 25]. Interestingly, the predominance of with-the-rule astigmatism in our older participants is less common in Western research, but it has been noted in some Asian populations. This suggests that ethnic or environmental factors might influence how the cornea ages. This study has practical implications for cataract surgery. Because corneal astigmatism is highly prevalent, it should be routinely assessed before surgery. Furthermore, surgical planning for patients with corneal astigmatism of ≥1.0 D should incorporate astigmatism correction. Patient counselling should include discussion of astigmatism correction options, particularly for those with moderate to high astigmatism. Training programs for cataract surgeons should emphasize astigmatism assessment and correction techniques. Several areas warrant further investigation based on our findings. Longitudinal studies tracking age-related changes in corneal astigmatism patterns would provide valuable insights into corneal aging in our population. Corneal astigmatism plays a critical role in postoperative refractive outcomes after cataract surgery, and advances in precise measurement, individualized surgical planning, and modern technologies such as toric IOLs and intraoperative aberrometry have greatly improved the accuracy of astigmatism correction and overall visual results [26]. Studies incorporating posterior corneal astigmatism measurements would provide more complete assessment of total corneal astigmatism. Outcomes studies comparing different astigmatism correction techniques in our population would help optimize surgical approaches. Cost-effectiveness analyses of routine astigmatism correction would inform healthcare policy decisions.

## Conclusion:

Corneal astigmatism is highly prevalent among cataract surgery candidates, highlighting its importance in pre-operative evaluation. Thus, addressing astigmatism during cataract surgery can significantly enhance visual outcomes and reduce dependence on spectacles. These findings highlight the need for routine astigmatism assessment and individualized surgical planning in modern cataract care.
